# The Immune Endocannabinoid System of the Tumor Microenvironment

**DOI:** 10.3390/ijms21238929

**Published:** 2020-11-25

**Authors:** Melanie Kienzl, Julia Kargl, Rudolf Schicho

**Affiliations:** 1 Division of Pharmacology, Otto Loewi Research Center, Medical University of Graz, Universitätsplatz 4, 8010 Graz, Austria; melanie.kienzl@medunigraz.at (M.K.); julia.kargl@medunigraz.at (J.K.); 2 BioTechMed, 8010 Graz, Austria

**Keywords:** tumor microenvironment, endocannabinoid system, cannabinoid receptors, immune cells

## Abstract

Leukocytes are part of the tumor microenvironment (TME) and are critical determinants of tumor progression. Because of the immunoregulatory properties of cannabinoids, the endocannabinoid system (ECS) may have an important role in shaping the TME. Members of the ECS, an entity that consists of cannabinoid receptors, endocannabinoids and their synthesizing/degrading enzymes, have been associated with both tumor growth and rejection. Immune cells express cannabinoid receptors and produce endocannabinoids, thereby forming an “immune endocannabinoid system”. Although in vitro effects of exogenous cannabinoids on immune cells are well described, the role of the ECS in the TME, and hence in tumor development and immunotherapy, is still elusive. This review/opinion discusses the possibility that the “immune endocannabinoid system” can fundamentally influence tumor progression. The widespread influence of cannabinoids on immune cell functions makes the members of the ECS an interesting target that could support immunotherapy.

## 1. Introduction

Gene mutations either caused by inheritance, environmental influence, faulty DNA replication or epigenetic modifications, and the accumulation and aberrant activity of these genes are key features in the process of cancer development [1,2]. Cells that aberrantly express these genes are constantly recognized and subsequently eradicated by cells of the immune system during tumorigenesis in a process called immune surveillance [3]. Nonetheless, mutated cells escape this process and succeed in developing cancer through the selection of tumor cell variants that either lack immunogenic features of recognition or exhibit features for the suppression of the evoked immune response [4].

Maintenance of tissue homeostasis is the work of immune cells, fibroblasts, the vasculature and extracellular matrix components. Apart from cancer cells, neoplastic lesions contain additional cell types, such as endothelial cells, pericytes, cancer-associated fibroblasts and immune cells [5]. Together, they can serve as a hurdle of cancer development [6]. Similar to inflammation, aberrant signaling, driven by cytokines and lipid mediators, among them also endocannabinoids, cause changes in tissue homeostasis and a shift towards a pro-tumorigenic environment and eventually to the development of cancer [6,7]. Thus, ongoing inflammation constitutes one of the hallmarks of cancer [5]. Like in inflammation, cells of the innate and adaptive immunity infiltrate tumors to form the immune tumor microenvironment (TME) with the aim to combat neoplastic growth [8]. Many of these cells express components of the endocannabinoid system (ECS), such as cannabinoid receptors [9,10,11,12]. Immune cells interact with each other and with tumor cells, they react to other components of the TME and the ECS, and they can subsequently halt but also contribute to tumor progression in experimental and clinical cancer [8,13]. All types of immune cells can be observed in tumors, including macrophages, dendritic cells (DCs), neutrophils, eosinophils, mast cells, natural killer (NK) cells, and B and T cells (including Th cells, and cytotoxic T cells) [8]. Importantly, disease-free and overall survival critically depends on the immune cell compositions within the TME [8].

## 2. The Endocannabinoid System (ECS)

Many immune cells contain components of the ECS, an entity that regulates organ- and cell-specific physiological events with the aim to restore cell and tissue homeostasis. It includes the cannabinoid receptors 1 and 2 (CB_1_ and CB_2_), the endogenous ligands of the cannabinoid receptors, the so-called endocannabinoids, such as anandamide (AEA) and 2-arachidonoylglycerol (2-AG), their enzymes for synthesis (diacylglycerol lipase (DAGL)), *N*-acylphosphatidyl-ethanolamine phospholipase D (NAPE-PLD) and degradation (fatty acid amide hydrolase (FAAH) and monoacylglycerol lipase (MGL)), and their transporters [14,15]. The ECS is widely expressed throughout the body and can be found in almost all organs, however, the human nervous system and the immune system have been found to represent the highest expression levels of cannabinoid receptors [16]. Parts of a wider ECS network are (i) non-cannabinoid receptors that show responsiveness to cannabinoids, such as G protein-coupled receptors 55 and 18 (GPR55, GPR18), PPAR receptors, TRP- and 5HT3receptors, potassium channels, and (ii) endocannabinoid-like lipids such as oleoyl- and palmitoyl-ethanolamide (OEA and PEA). These components belong to an expanded ECS (endocannabinoidome; [17]). See Pertwee [18] and Cristino et al. [15] for a more detailed description of the ECS and the “endocannabinoidome”.

## 3. The Endocannabinoid System and the Tumor Microenvironment

This review discusses the potential role of (endo)cannabinoids and other ECS components in immune cells that are typically found in the TME. For detailed effects of cannabis/(endo)cannabinoids on tumor cells and cannabinoid receptor signaling, the reader is referred to several other recent reviews [19,20,21,22].

Receptors and enzymes of the ECS have been mostly measured and quantified by immunohistochemical, Western blot and PCR methods using tissue from a variety of tumor models and biopsies from patients with, e.g., breast, brain, prostate, colon and cervical cancer. Each of the tumors may exhibit either up- or down-regulation of cannabinoid receptors (which are often increased in tumors), and of endocannabinoids and their metabolizing enzymes, FAAH and MGL (rev. in [20]). Correlations between expression of cannabinoid receptors and disease outcome largely differ between various types of cancer [20] indicating that there is no universal (e.g., anti-carcinogenic) role of the ECS in tumor development but that its role rather depends on the type of the tumor. For instance, CB_2_ overexpression in HER-2 positive breast cancer is a marker for poor outcome [23], whereas in hepatocarcinoma, CB_1_ and CB_2_ expression correlate with good clinical outcome [24].

(Endo)cannabinoids have direct anti-carcinogenic effects on tumor cells [19,25,26]. These effects include inhibition of proliferation, cell cycle arrest, apoptosis and autophagy [26,27]. Thus, AEA- and 2-AG-dependent anti-proliferative effects have been demonstrated in colon, breast, prostate and cervical cancer cells [20,28,29]. Many of these studies were also conducted with exogenous cannabinoids such as Δ^9^-THC, which mimics the effects of endocannabinoids on cannabinoid receptors [19]. In this context, however, Δ^9^-THC has shown biphasic effects, inducing cancer cell growth at low (100–300 nM) [30] and cell death at high (µM) concentrations [31].

**Figure 1:**
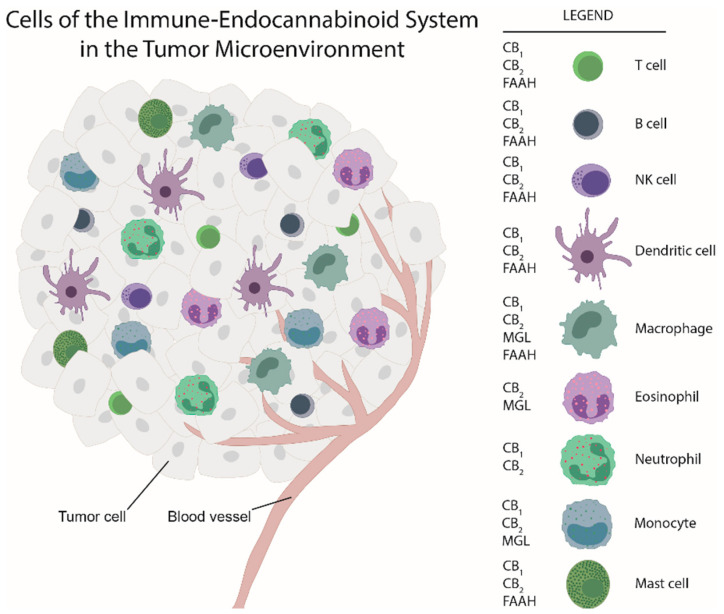
Tumors locally recruit immune cells that reject and also promote tumor development and metastasis. Immune cells express components of the endocannabinoid system, thereby they are able to form an “immune endocannabinoid system” within the tumor microenvironment. Most of the immune cells express cannabinoid receptors (CB_1_, CB_2_) and enzymes for endocannabinoid degradation (monoacylglycerol lipase (MGL) and fatty acid amide hydrolase (FAAH)). In addition, they are known to release endocannabinoids (see Table 2).

While there is ample evidence that cannabinoids and components of the ECS are involved in inhibiting tumor cell proliferation in vitro, little is known about the impacts the ECS has on cells of the TME and consequently on tumor progression. A study by Busch et al. demonstrated that in models of lung adenocarcinoma with different types of mutation (in Kras, p53, or Egfr), the immune cell content varied, suggesting that immune responses and TME landscape of tumors critically depend on tumor cell mutations [32]. As for the ECS, its components are located in immune cells (see Figure 1) besides their expression in tumor cells. Among the few studies that have addressed the ECS in the TME, our group showed, by use of a chemically induced colorectal cancer model, a marked shift in the composition of the immune TME in GPR55 knockout vs. wildtype mice. Knockouts displayed a lower amount of MDSCs which suppress anti-tumor immunity [33], but a higher number of CD4^+^ and CD8^+^ cells (which correlate with better prognosis) [34]. Among the other studies, Qiu et al. reported that 2-AG induced the expansion of MDSCs in a model of pancreatic adenocarcinoma with no effect on CD4^+^ and CD8^+^ cells [29]. In a model of colon cancer with mice bearing MGL-deficient macrophages, a lower tumor burden was observed in knockouts as compared to wildtypes in a study by Xiang et al. (2018) [35]. Zhu et al. demonstrated that Δ^9^-THC suppressed host immune reactivity to lung cancer via inhibitory cytokines [36].

To date, these data suggest that exogenous cannabinoids and ECS components have an influence on immune cells of the TME and that the ECS could be involved in the control of this immune cell network and hence in tumor growth.

Before we discuss ECS components of immune cells and their relation to the TME, a brief introduction of the role of immune cells (expressing ECS components) in the TME is given in the following sections.

## 4. Immune Cells in the Tumor Microenvironment

### 4.1. T Lymphocytes

#### 4.1.1. CD8^+^ T Cells

Tumors are infiltrated by various T cell populations that preferentially reside in the invasive tumor margin and the draining lymphoid organs [8]. Among these populations, CD8^+^ T cells are capable of detecting tumor cells via recognition of aberrant antigens from overexpressed or mutated molecules that are presented by major histocompatibility complex I (MHC I) [37]. After antigen and MHC I recognition, cytotoxic molecules, such as granzymes and perforin, are released by CD8^+^ T cells and result in tumor cell killing [38]. Other mechanisms that underlie the killing of tumor cells via CD8^+^ T cells include the death receptors TRAIL and FasL (reviewed by Martínez-Losato and colleagues [39]). Increased numbers of CD8^+^ T cells in tumors are associated with a better clinical outcome in patients, e.g., with breast [40,41] and colorectal cancer [42,43], and glioblastoma [44]. However, anti-tumorigenic lymphocytes may become exhausted or dysfunctional due to the engagement of effector molecules or inhibitory receptors (e.g., T cell immunoglobulin domain and mucin domain protein 3 (TIM-3), cytotoxic T lymphocyte antigen-4 (CTLA-4) and T cell immunoglobulin and immunoreceptor tyrosine-based inhibitory motif domain (TIGIT)) of tumor and TME cells [45,46]. Furthermore, engagement of programmed cell death protein ligand-1 (PD-L1) on tumor cells with programmed cell death protein-1 (PD-1) expressed on CD8^+^ T cells reduces the susceptibility of tumor cells to T cell-mediated killing, inducing tumorigenesis [47]. In recent years, antibodies targeting those inhibitory molecules and their ligands were moving into the focus of immunotherapies, namely as immune checkpoint inhibitors (ICI), with CTLA-4 and PD-1 being the most successful targets (reviewed in [46]).

#### 4.1.2. CD4^+^ T Cells

The infiltration of different CD4^+^ T cell subpopulations has been described in solid tumors [48,49]. CD4^+^ T helper 1 (Th1) cells mediate anti-tumor effects with the help of CD8^+^ T cells [50], hence, elevated numbers of CD4^+^ T helper 1 cells in the TME correlate with a positive clinical outcome in various human tumors (rev. in [8]).

For other T helper cell populations, e.g., Th2, Th17 or Th22, a role in the TME and in tumor growth has been suggested, however, the effects on tumor development are contradictory [8,51,52,53,54].

Other immunosuppressive CD4^+^ T cells, i.e., regulatory T cells (Tregs), are often described as pro-tumorigenic (reviewed in [8]). Elevated numbers of Tregs inversely correlate with the survival of patients with ovarian [55] and breast cancer [56], and hepatocellular carcinoma [57], although the opposite was observed for follicular [58] and Hodgkin’s lymphoma [59]. Mechanisms linked to the suppressive function of Tregs include secretion of suppressive cytokines, cytolysis of effector T cells, metabolic disruption, and DC suppression (reviewed in [60]).

### 4.2. B Lymphocytes

B cells are important cells of the humoral immunity and infiltrate tumors where they mostly localize to tertiary lymphoid structures (TLS) [61]. B cell-mediated tumor cell killing can be directly accomplished via the Fas/FasL or TRAIL/Apo2L pathways [62,63], or indirectly via production of IFN-γ [64], thus recruiting and activating NK cells and polarizing T cells towards Th1 [61]. Another anti-tumorigenic effect of B cells is mediated by anti-tumor antibodies, as recently reviewed by Sharonov et al. [61]. Cancer-specific neo-antigens, such as mutated p53 [65], but also self-proteins represent targets for antibodies in tumors [66,67]. Infiltrating B cells were reported as an important predictor for good clinical outcome in patients with metastatic melanoma [68]. Further studies identified a correlation of peritumoral B cells [69] and infiltrating B cells [70,71] with reduced relapse rates and prolonged survival in cervical and lung cancer, respectively.

However, B cells and antibody production may not be only associated with less tumor growth. As to the prognostic role of anti-p53 antibodies, contradictory results were reported in cancer patients [72]. Antibody production in melanoma-draining lymph nodes accelerated tumor growth, although this was attenuated by macrophages [73]. Not only antibodies may promote tumor growth, but also an immunosuppressive subpopulation of B cells (regulatory B cells) [61]. This population assists the generation of Tregs [74,75] and expresses inhibitory ligands [76] and cytokines (i.e., IL-10 [77]), resulting in inhibition of CD4^+^ and CD8^+^ T cells and the promotion of tumor growth [78]. It is, therefore, not surprising that B cells have been reported to be a sign for negative overall survival in various human cancer types such as, bladder, breast, and colorectal cancer, amongst others (reviewed in [61]).

### 4.3. NK Cells

NK cells are part of the innate immune system and eliminate cells with aberrant or absent expression of MHC I [79], a mechanism used by cancer cells to escape recognition by CD8^+^ T cells [80]. Killing of tumor cells by NK cells is mediated by the release of lytic granules that contain perforin and granzymes, resulting in tumor cell apoptosis [81]. Death receptor-mediated killing of tumor cells via TRAIL and FasL is also harnessed for cancer cell elimination [82]. Generally, NK cells predict a good prognosis for many solid tumors [83], while sparse NK cell function was reported to correlate with the development of metastases in pharyngeal [84], head and neck [85] and other solid tumors [86]. An anergic/exhausted phenotype of NK cells has been reported in the stroma of lung [87] and colon cancer [88]. NK cell may well depict a target for cancer immunotherapy strategies, as recently reviewed [89].

### 4.4. Neutrophils

Until recently, neutrophils were only regarded as bystanders in cancer [90], however, they constituted 20% of all CD45^+^ cells in non–small-cell lung cancer (NSCLC) tumor specimens, thus representing a major immune cell type in NSCLC [91]. They also represent a considerable portion of the infiltrating immune cells in other cancer types [92]. In NSCLC, neutrophil infiltration results in depletion of CD4^+^ and CD8^+^ T cells [93]. Clinical studies established a pro-tumorigenic role of tumor-associated neutrophils (TANs), for instance, in renal cell carcinoma [94], colorectal cancer [95], and cervical cancer [96]. In these studies, infiltrated neutrophils were associated with poor survival, thus, they were suggested to support the development of a malignant phenotype. In contrast, elevated numbers of TANs in stage II colorectal cancer resulted in improved survival [97]. TANs were also reported to be a favorable prognostic factor in gastric and colorectal cancer [98,99]. The role of neutrophils, therefore, in the TME seems contradictory, suggesting a certain plasticity between a pro- (N2-neutrophils) and anti-tumorigenic (N1-neutrophils) state [100]. Thus, Zhang and Houghton proposed TANs as targets for immunotherapy [101], and to either drain TANs completely or to focus on the pro-tumorigenic molecules secreted by TANs. In fact, the use of SX-682, a CXCR1/2 inhibitor, proved to be beneficial in a combined treatment regimen with ICI therapy in a mouse model, suggesting enhanced efficacy of ICI by neutrophil antagonism in NSCLC patients [93].

### 4.5. Eosinophils

Eosinophils are important immune regulators [102]. As such, it is likely that eosinophils can fundamentally shape the TME (rev. in [103,104]). Since they show pro- as well as anti-tumorigenic effects, Varricchi et al. suggested that different tumor entities accompanied with differences of the surrounding milieu affect the function of tumor-associated eosinophils [103]. Eosinophilia of blood and tumors are favorable for the outcome of several types of cancer (rev. in [103]). In experimental tumors, activated eosinophils either reduce tumor growth directly via degranulation [105,106] or, additionally, via recruitment of other anti-tumorigenic leukocytes [107,108]. In contrast to their anti-tumorigenic features, an association with tumor growth was also reported (rev. in [103]). Given that eosinophils are regular cells of the TME, they should be considered as important players that may likely influence immunotherapy of cancer patients [104].

### 4.6. Mast Cells

Mast cells belong to the innate immune system and contribute to various diseases, including cancer [109]. Infiltration of mast cells has been suggested to be either anti- or pro-tumorigenic in clinical cancer (rev. in [7]). In experimental tumor models, the role of mast cells is also contradictory [7]. Interestingly, tumor progression was unaffected by the presence of mast cells in colorectal [110], and renal cancer [111]. In summary, studies suggest a cancer-specific role for mast cells and their effector molecules, but still many questions need to be answered before mast cells can be considered as a therapeutic target [7].

### 4.7. Monocytes

Monocytes are cells of the innate immune system, circulating in the blood before trafficking into the tissue. They maintain tissue homeostasis, support immunity, and suppress excessive immune responses [112]. Under pathological conditions, recruitment of monocytes to the site of inflammation and tumors is enhanced [113]. Recent studies described conflicting roles of monocyte subsets in cancer: on the one hand, inflammatory monocytes promote tumor growth [114] and correlate with poor clinical outcome [115], but, on the other hand, patrolling monocytes can hamper the development of metastases [116]. Monocytes trafficking into tumors differentiate either into DCs, especially into monocyte-derived DCs (moDCs) or into tumor-associated macrophages (TAMs) [113,117]. They can also maintain their state and be part of a monocyte pool [118].

### 4.8. Tumor-Associated Macrophages (TAMs)

TAMs were identified in the stroma of various types of cancer [117]. Elevated numbers of TAMs were reported to be linked with poor prognosis in human cancers, such as gastric, breast, bladder, and ovarian cancer, and Hodgkin’s lymphoma [119,120]. Experimental studies, which support these clinical findings, revealed mediators secreted by TAMs as driving factors of tumor progression, either directly or indirectly [117]. Thus, VEGF-A produced by TAMs was shown to regulate angiogenesis and tumor progression in the PyMT (polyoma middle T oncoprotein) mouse model of breast cancer [121]. Other mechanisms include the rearrangement of the tumor architecture (e.g., via MMPs [122]), the induction of tumor cell proliferation and survival (e.g., through epidermal growth factor [123] or IL-6 [124]), and the suppression of anti-cancer immunity (e.g., via PD-L1 [125]). On the other hand, elevated presence of TAMs in colon [119,126], gastric [127] and nests of endometrial cancer [128] was linked to improved survival in clinical studies.

The influence of TAMs on tumor development may be better understood via the identification of their functional state [117]. Thus, macrophages are broadly sub-classified into M1 (classically activated) and M2 (alternatively activated) cells, differing in the cytokines needed for their polarization and effector functions [129]. M1-polarized macrophages have been reported to exert anti-tumorigenic functions. On the contrary, M2 macrophages are associated with immunosuppression [130], as well as with increased secretion of molecules for tissue remodeling, repair and angiogenesis [121,131]. Given the different functions of macrophage subtypes in tumor development, macrophages depict an attractive target for immunotherapies, either via depletion or via reprogramming towards anti-tumorigenic activities (reviewed in [132]).

### 4.9. Dendritic Cells (DCs)

DCs are antigen-presenting cells which screen their environment for antigens, followed by activation of cells of the adaptive immunity [133]. Increased infiltration of classic DCs (cDCs) into the TME is associated with a better disease outcome in lung cancer and melanoma [134,135,136]. Accumulation of plasmacytoid DCs (pDCs), another DC subset, has been reported in cancer, however, infiltration of this subset was associated with poor survival in breast cancer [137] and melanoma [94].

### 4.10. Myeloid-Derived Suppressor Cells (MDSCs)

MDSCs are of neutrophilic (PMN-MDSC) or monocytic origin (M-MDSC), and they are mainly detected in patients (or animals) with ongoing inflammation, infection or cancer [138,139]. MDSCs exert their suppressive function via various pathways. For example, they have been reported to induce the development of Tregs and the suppression of T cells [140,141]. M-MDSCs may also differentiate into TAMs and inflammatory DCs in the TME [142]. Furthermore, they may promote tumor development by enhancing cancer stemness [143] and angiogenesis [144]. It is, therefore, not surprising that increased frequencies of circulating MDSCs have been associated with poor disease prognosis in breast cancer melanoma patients [145].

## 5. Cells of the “Immune Endocannabinoid System”

Many, if not all, immune cells express cannabinoid receptors and are able to produce and degrade endocannabinoids (rev. in [146]; Figure 1; actions of (endo)cannabinoids and cannabinoid receptor ligands on these immune cells are summarized in Table 1).

Therefore, the ECS members virtually constitute an “immune endocannabinoid” system ([147]; see Table 2 for ECS member expression in immune cells). In a study by Galiègue et al., human leukocytes were shown to express CB_2_ receptors at the following levels: B cells > NK cells > PMN >neutrophils > CD8^+^ T cells > monocytes > CD4^+^ T cells, however, significantly less (or no) expression of CB_1_ was observed in these cells [16], indicating a regulatory role in immune cell function primarily for CB_2_ [148]. It should be noted though that expression levels of cannabinoid receptors may vary in these cells depending on the cells’ localization and environment. The presence of inflammatory cytokines (IL-6, IL-1β and TNF-α), for instance, was shown to increase CB_1_ and CB_2_ expression in peripheral blood mononuclear cells [149]. Since these cytokines are present in tumors and often increased (promoting growth; [150]), cannabinoid receptor expression in immune cells may be significantly higher in tumors than in healthy tissues. In line, tumors of the brain, prostate, pancreas and cervix all show higher levels of CB_1_ and CB_2_ receptors as compared to normal tissue [20].

**Table 1:** Some main actions of (endo)cannabinoids and cannabinoid receptor ligands on immune cells.

Immune Cells	Effects of (Endo)Cannabinoids or Synthetic Cannabinoid Receptor Ligands	(Endo)Cannabinoids/Ligands	Reference
T cells (human, mouse)	Inhibition/induction of Th1 and Th2 cytokines	Δ^9^-THC	[151,152,153,154]
T cells (human)	Suppression of proliferation and cytokine release via CB_2_ Induction of apoptosis Inhibition of migration	AEA AEA AEA	
B cells (human)	Stimulation of migration Inhibition of proliferation	2-AG AEA	[153,155,156,157]
B cells (mouse)	Stimulation of migration	2-AG	
NK cells (human)	Stimulation of migration via CB_2_	2-AG	[158]
Dendritic cells (human)	Inhibition of cytokine production in myeloid and plasmacytoid dendritic cells	AEA	[159,160]
Dendritic cells (mouse)	Inhibition of Th1 and Th17 lineage induction Stimulation of migration	AEA 2-AG	
Macrophages (mouse)	Stimulation of ROS production via CB_1_ Inhibition of TNF-α production Suppression of ROS	AEA, ACEA 2-AG 2-AG	[161,162,163,164,165]
Macrophages (human)	Inhibition of migration via CB_2_ Rapid actin polymerization via CB_2_ Stimulation of migration	Δ^9^-THC 2-AG 2-AG	
Eosinophils	Stimulation of migration via CB_2_	JWH133, 2-AG	[166,167]
Neutrophils (human)	Activation (MPO release, Ca^++^ mobilization) Suppression of migration No effect on migration	2-AG JWH015, 2-AG Δ^9^-THC	[168,169,170]
Mast cells (human)	Control of degranulation via CB_1_	AEA, ACEA	[171]

2-AG, 2-arachidonylglycerol; AEA, anandamide; ACEA, arachidonyl-2′-chloroethylamide (CB_1_ agonist); CB_1_, cannabinoid receptor 1; CB_2_, cannabinoid receptor 2; Δ^9^-THC, delta 9-tetrahydrocannabinol; MPO, myeloperoxidase; ROS, reactive oxygen species; TNF-α, tumor necrosis factor alpha; JWH015 and JWH133 are CB_2_ agonists.

**Table 2:** The immune endocannabinoid system.

	CB_1_ Receptors (Species; Method of Detection)	CB_2_ Receptors (Species; Method of Detection)	MGL (Species; Method of Detection)	FAAH (Species; Method of Detection)	Production of Endocannabinoids
PBMC	-human; PCR, FC, WB; T cells activated with TNFalpha [149] -human; PCR; [172] -human; PCR; [161]	-human; PCR, FC, WB; T cells activated with TNF-α; [149] -human; PCR; CB_2_ 3 x higher than CB_1_; [172] -human; PCR; [161]			
Lymphocytes	-human; PCR, WB [173]			-human; ELISA, PCR [173]	AEA [173] 2-AG [174]
T cells	-human; PCR; T cells activated with CD3/28; [175] -human; PCR; T cells activated with TNF-α; [149] -human; PCR; [176]	-human; PCR; T cells activated with CD3/28; [175] -human; PCR [16] -human; PCR; T cells activated with TNFalpha [149] -human; FC; [177] -human; PCR; [176]		-human; PCR; [176]	
B cells	-human; PCR; [176]	-human; PCR; [16] -human; FC; [177] -human; FC; [178] -human; PCR; [176] -human, FC; [179]		-human; PCR; [176]	
Monocytes	-human THP monocytes; PCR; [161]	-human; PCR; [16] -human THP monocytes; PCR; [161] -human, FC; [179]	-human; WB; [180]		2-AG [174]
Macrophages	-human PMA-treated monocyte-derived macrophages; PCR; [161] -mouse RAW264.7 cells; PCR; [161] -human PBMC-derived macrophages; PCR; [181] -rat; circulating macrophages; PCR; [182]	-human; PCR; [16] -human PMA-treated monocyte-derived macrophages; PCR; [161] -mouse RAW264.7 cells; PCR; [161] -human; differentiated monocytes; PCR; [183] -mouse tumor-associated macrophages; PCR; [35]	-mouse; tumor associated macrophages; [35]	-rat; circulating macrophages; PCR; [182]	AEA in RBL-2H3 basophils, J774 and RAW264.7 mouse macrophages [182,184,185,186] 2-AG in mouse peritoneal macrophages [187] 2-AG in J774 cells [182] 2-AG in mouse P388D1 macrophages [188] 2-AG in mouse peritoneal macrophages [189] 2-AG in RAW264.7 cells [186]
NK cells	-human; PCR; [176]	-human; PCR; [16] -human; FC; [177] -human; PCR; [176] -human, FC; [179]		-human; PCR; [176]	
Dendritic cells	-human; PCR, WB; [190]	-human; PCR, WB; [190]		-human; PCR, WB; [190]	AEA, 2-AG [190]
Neutrophils	-mouse bone marrow neutrophils (liver injury model); PCR; IF; [191]	-human; PCR; [16] -human; FC; [169] -not detected [168] -mouse bone marrow neutrophils (liver injury model); PCR; IF; [191] -human; WB; [192] -human, FC; [179]			2-AG [174]
Eosinophils		-human; PCR; [168] -human; PCR, Northern Blot; [167] -human; FC, [166]	-human; PCR; [168]		2-AG [174]
Mast cells	-rat RBL2H3 cells; PCR; [193] -mouse (primary BMMCs); WB; [194] -human mucosal-type mast cells; IHC; [171]	-rat RBL2H3 cells; PCR; [193] -mouse (primary BMMCs); WB; [194]		-human mast cells (HMC-1); FAAH activity measured; [195]	

AEA, anandamide; 2-AG, 2 arachidonoylglycerol; BMMC, bone marrow-derived mast cell; CB_1_, cannabinoid receptor 1; CB_2_, cannabinoid receptor 2; ELISA, enzyme-linked immunosorbent assay; FAAH, fatty acid amide. hydrolase; FC, flow cytometry; IF, immunofluorescence; IHC, immunohistochemistry; MGL, monoacylglycerol lipase; PBMC, peripheral blood mononuclear cells; PCR, polymerase chain reaction; PMA, phorbol 12-myristate. 13-acetate; WB, Western blot.

### CB_1_ and CB_2_ Receptors in Immune Cells

As it can be seen from Table 2, most information on ECS components in immune cells is available for CB_1_ and CB_2_ receptors. They are expressed in almost all types of immune cells, suggesting that the inflammatory behavior of these cells is regulated through endocannabinoid activation. Both receptors couple to Gi/o proteins and inhibit adenylyl cyclase, but show some differences in other downstream effects. For instance, CB_2_ receptors do not couple to potassium channels, such as the G-protein-gated inwardly rectifying potassium (GIRK) channels, providing an explanation as to its functional difference to CB_1_ (rev. in [196]). In inflammatory conditions, CB_1_ as well as CB_2_ receptors may show equal actions, e.g., reduction of VEGF-A secretion in LPS-stimulated neutrophils [197] and human lung-resident macrophages [198]. However, CB_1_ and CB_2_ activation may also have opposing effects, especially with regard to reactive oxygen species (ROS) production and polarization of macrophages. In macrophages, activation of CB_1_ enhances production of ROS and TNF-α, while activation of CB_2_ suppresses these effects [161]. Additionally, CB_1_ induces polarization towards an M1 phenotype [199], whereas activation of CB_2_ causes a shift towards an anti-inflammatory M2 phenotype [200].

The role of CB_2_ in inflammation and immune cell function is well described, but unanswered questions remain as to whether activation of CB_2_ is anti- or pro-inflammatory [196]. For instance, activation of CB_2_ by AEA reduces proliferation and inhibits release of pro-inflammatory cytokines, such as IL-2, TNF-α and IFN-γ, from primary T-lymphocytes; furthermore, IL-17 secretion from Th17 cells is suppressed [152]. In addition, CB_2_ decreases expression of inflammatory cytokines [201] and accumulation of oxLDL in macrophages [202], indicating anti-inflammatory actions of CB_2_. On the other hand, migration of B cells, dendritic cells and eosinophils [156,160,166,167], and adhesion of monocytes and macrophages is increased by 2-AG [164,165,203], also suggesting pro-inflammatory actions of CB_2_.

In vivo effects of cannabinoid receptors have been mostly associated with anti-inflammation. However, pro-inflammatory in vivo effects of endocannabinoids have been also described, suggesting that actions of endocannabinoids (and especially of their metabolites) on cannabinoid receptors, and also the role of non-CB_1_/CB_2_ cannabinoid-related G protein-coupled receptors in inflammation are not yet fully understood (rev. in [146,204]).

## 6. The “Immune Endocannabinoid System” in Adaptive and Innate Immunity

From in vitro/vivo experiments with exogenous cannabinoids, there is solid evidence that cannabinoids influence key functions of immune cells, such as proliferation, migration, antibody formation, cytolytic activity, differentiation and apoptosis (rev in [146,147,151,205,206,207]. All of these functions are relevant for the immune cell composition of the TME and tumor growth.

Among the adaptive immune system, T cells are particularly influenced by cannabinoids, repeatedly shown by in vitro experiments using Δ^9^-THC as an agent [147]. Thus, strong influence on proliferation of T cells by cannabinoids has been demonstrated in vitro [153] as well as in vivo [147]. In particular, regulation of CD8^+^ T cell function, which, owing to their tumoricidal activity, critically determine cancer growth, is of importance. Together with CD4^+^ Th1 cells, they are associated with good disease prognosis [208,209]. Differentiation of T cells may represent another function influenced by the ECS in the TME. A previous study showed that perinatal exposure to Δ^9^-THC caused a marked alteration in T cell subpopulations, which was dependent on CB_1_ and CB_2_ [210]. In line with the cannabinoids’ effects on T cell function and development, Yuan et al. demonstrated that Δ^9^-THC regulates Th1/Th2 cytokine balance in activated human T cells [211]. B cells are another immune cell population whose differentiation depends on ECS components such as CB_2_ receptors [177,178].

Therefore, CD8^+^, CD4^+^, and B cells could be highly susceptible to the effects of endocannabinoids within the TME, owing to their expression of CB_2_ and CB_1_ receptors (see Table 2).

Marijuana is well known for its immunosuppressive effects [212]. For instance, Δ^9^-THC has been shown to worsen Legionella, herpes simplex and Listeria infections (rev. in [146,213] and to lower the number of T cells in mice after daily treatment (s.c.,10 mg/kg for 14 days) [214]. In addition, in humans, cell-mediated immunity and host defense is suppressed by Δ^9^-THC [215]. A study in cannabis users revealed a reduction in lymphocyte functionality and NK cell number, and a disruption of the Th1/Th2 balance [216], which could be associated with increased infection and impairment of cytokine production. Smoking of cannabis is also connected with alterations in the basal levels of CB_1_ and CB_2_ from PBMCs [172].

In line with their immunosuppressive role, cannabinoid ligands have been demonstrated to suppress phagocytosis, cell spreading, antigen presentation and other features of immune cells [217,218,219,220], all of which are essential for immune cell regulation in the TME. CB_2_ receptor agonists, in particular, cause immunosuppression (rev. in [221]) as highlighted by a study from Zhu et al. who showed that activation of cannabinoid receptors by Δ^9^-THC inhibited anti-tumor immunity through an CB_2_-mediated increase in tumor promoting cytokines [36]. However, CB_2_ may have multiple roles in the TME since the receptor can stimulate migration of myeloid leukemia cells and normal splenocytes [156]. Furthermore, CB_2_ activates macrophages [35] and induces apoptosis in immune cells [222].

Certain innate immune cells of the TME, including neutrophils, M2 macrophages and MDSCs, have been associated with tumor progression [208]. Since ECS components are expressed in innate immune cells (see Table 1), endocannabinoid signaling in these cells may influence functions relevant for tumor growth. Macrophages are highly responsive to cannabinoids in terms of cytokine secretion, migration, phagocytosis and antigen presentation (rev. in [151]). They express cannabinoid receptors [35,181,183] and MGL [35,223], and a CB_2_ dependent pro-tumorigenic role of macrophage-expressed MGL has been recently described [35]. In addition, Δ^9^-THC was shown to inhibit Th cell activation through macrophages derived from CB_2_ wildtype, but not from CB_2_ knockout mice [224], indicating that macrophages and CB_2_ are important in directing T cell responses.

Neutrophils exert immunosuppressive properties [225] and are the most prevalent immune cell type in non–small-cell lung cancer [91]. The gene signature of neutrophils predicted mortality better than any other immune cell signature in a cohort of >18k patients [226]. Neutrophils express CB_2_ receptors [16]; the receptors suppress migration [169] and inhibit cell differentiation when overexpressed in myeloid precursor cells [227]. Hedge et al. recently showed that activation of cannabinoid receptors with Δ^9^-THC mobilized MDSCs (which contain various forms of PMNs) and led to immunosuppression [228]. It was previously demonstrated that CB_2_ (cooperating with GPR55) is involved in human neutrophil function [192]. The precise role of the ECS in tumor-associated neutrophils, however, remains elusive.

Dendritic cells (DCs) are also integral part of the TME and are essential in staging an adaptive immune response [208]. These cells have now moved into the light of anti-tumor therapy as their presence may promote susceptibility to immunotherapy [229]. Human DCs express CB_1_ and CB_2_ receptors and also produce AEA and 2-AG [146], suggesting potential effects on the TME. CB_2_ has been recently reported to influence dendritic cell maturation [230]. Another innate immune cell population with cannabinoid receptor expression is NK cells [16], which are well linked to tumor regression [231]. Cannabinoids such as Δ^9^-THC are able to suppress NK activity [232] which may involve both CB_1_ and CB_2_ receptors [233].

To summarize, regulation of T cells, macrophages and dendritic cells by endocannabinoids and MGL is a potential mechanism by the ECS to control tumor growth. This could be primarily achieved by CB_2_ activity, although CB_1_ and cannabinoid receptor-independent mechanisms may also be involved (rev in [234]). The role of the ECS in neutrophils awaits exploration. For additional reading about cannabinoids and immunity (not only related to cancer), the reader is referred to the following reviews: [206,235,236].

## 7. Potential Role of Endocannabinoids in the Tumor Microenvironment

Although alterations in endocannabinoid levels have been demonstrated in tumors vs. non-neoplastic tissue [237], a clear understanding of how endocannabinoids impact the immune TME is hardly known. As to the role of endocannabinoids within the TME, their actions are likely dependent on their local concentration, status of immune cell activation and expression levels of cannabinoid receptors. As shown by Sailler et al. [237] and by our own studies [223], endocannabinoid profiles are deranged in tumors and plasma of cancer patients in comparison to control tissue/plasma. In many tumors, levels of 2-AG and AEA are increased, such as in brain, intestinal and gynecological tumors (summarized in [20]), whereas one study shows increases in 2-AG, but decreases in AEA, PEA and OEA in mice with local tumor growth [237], suggesting a consistent role of endocannabinoids in cancer only for 2-AG.

As in the case of immune cells, endocannabinoids were shown to suppress proliferation of T cells, migration of CD8^+^ cells and neutrophils, and the release of proinflammatory cytokines (e.g., TNF-α, IL-1β, IL-6, IL-17 and IFN-γ) from CD8^+^, CD4^+^, and dendritic cells and from macrophages (summarized and discussed in [146]). 2-AG has been described to cause migration of human peripheral blood monocytes, neutrophils, eosinophils, NK cells, mouse dendritic cells, and B cells [156,157,158,160,165,167,169]. In a pancreatic tumor model, 2-AG caused an increase in MDSCs, indicating immunosuppressive effects in vivo [29]. Adding to the endocannabinoids’ complex behavior, their effects may not be always mediated by cannabinoid receptors: Chouinard et al. demonstrated that 2-AG activated human neutrophils independently of cannabinoid receptors by a mechanism that includes 2-AG hydrolysis, de novo LTB_4_ biosynthesis, and an autocrine activation loop involving LTB_4_ receptor 1 [168]. 2-AG may also serve as a substrate for cyclo-oxygenase (COX)-catalyzing PGE_2_ production (e.g., via liberation of arachidonic acid by other hydrolases such as MGL), which in turn could modulate the action of CB_2_ [238]. In the TME, immune cells like macrophages could be a source of endocannabinoids. Their synthesis may even be stimulated by endocannabinoids themselves [185]. Macrophages such as mouse peritoneal macrophages [187], J774 [182] and P388D1 macrophages [188] were all shown to synthesize 2-AG. Furthermore, AEA is formed in macrophages [239] and was shown to maintain the level of regulatory macrophages (high expression of chemokine receptor CX3CR1) in gut tissue [240], supporting the idea that also in the TME, macrophage behavior is likely regulated by endocannabinoids.

It should be kept in mind that many effects of cannabinoids have been measured by in vitro experiments, and the effects were dependent on the concentration of the (endo)cannabinoids applied. Berdyshev and colleagues demonstrated that while AEA diminished IL-6 and IL-8 production in PBMCs at low nanomolar concentrations (3–30 nM), these effects disappeared after increasing the concentration [241]. Likewise, cannabinoids such as Δ^9^-THC, and the synthetic cannabinoid receptor ligands WIN 55212-2 and CP55,940, enhanced proliferation of human B cells at low (nM range), but not at high concentrations (10 µM) [242]. Since tissue concentrations of endocannabinoids lie in the nM range/g tissue (e.g., in colon mucosal tissue; [243]), effects seen with high µM concentrations in vitro may not be relevant for in vivo (patho)-physiology of immune cells. In vivo actions of endocannabinoids in the TME, therefore, could differ from those known from in vitro experiments.

To summarize, plenty of data show that endocannabinoids concentration-dependently influence immune cell behavior, a situation that may likely occur in the TME. The potential role of endocannabinoids in primary tumors and metastases has also been recently discussed elsewhere [244].

## 8. Cannabinoids as Potential Drugs That Affect the Tumor Microenvironment and Tumor Growth

The fact that cannabinoids can influence the immune cell behavior and, therefore, also the immune cell infiltrate of tumors (and hence tumor growth), naturally raises the suggestion that cannabinoid-based drugs could be used as anti-tumor agents alone or in combination with immunotherapy. Cannabinoid receptors are expressed on immune cells and cannabinoids may, therefore, modulate anti-tumor immune responses. Presently, cannabis and synthetic cannabinoids, such as nabilone, are mainly used as antiemetics during chemotherapy. Although preclinical models have demonstrated potent anticancer effects [245], sufficient evidence for the use of cannabis in cancer only exists in palliative care but not in anti-tumor therapy [246]. Δ^9^-THC has shown immunosuppressive effects and causes an increase in tumor growth in a breast cancer model [247], suggesting that the immune system does not favorably respond to cannabinoid treatment in cancer. Clinical studies also report that cannabis use decreased the response rate in cancer patients during immunotherapy with the PD-1 inhibitor nivolumab [248], and correlated with poor clinical outcome [249]. These results may speak against cannabinoids as add-ons in immunotherapy. However, it is still unclear how pharmacological inhibition of cannabinoid receptors or other targets of the ECS may affect immunotherapy in preclinical models of cancer.

## 9. Conclusions

In vitro studies have demonstrated that the behavior of immune cells is regulated by (endo)cannabinoids and other components of the ECS, indicating that the ECS effectively influences the immune landscape of tumors. This has been now supported by in vivo studies highlighting the importance of macrophages and MDSCs of the TME in the actions of the ECS on tumor growth (e.g., [29,34,35]). ECS components of the TME could be responsible for the fate of tumor growth by working synergistically, independently or in an opposing manner. Knowledge on the role of the ECS in the regulation of the “tumor immune microenvironment” may be important in establishing a more effective anti-neoplastic therapy.
